# Efficacy of endoscopic ultrasound‐guided abscess drainage for non‐pancreatic abscesses: A retrospective study

**DOI:** 10.1002/jgh3.12931

**Published:** 2023-06-19

**Authors:** Tomohiro Tanikawa, Mayuko Kawada, Katsunori Ishii, Noriyo Urata, Ken Nishino, Mitsuhiko Suehiro, Miwa Kawanaka, Ken Haruma, Hirofumi Kawamoto

**Affiliations:** ^1^ Department of General Internal Medicine 2 Kawasaki Medical School Okayama Japan

**Keywords:** abscess, drainage, endoscopic ultrasound‐guided abscess drainage, plastic stent

## Abstract

**Background and Aim:**

Percutaneous drainage of intra‐abdominal abscesses is often uncomfortable for the patient and may result in prolonged hospital stays. Recent studies have shown that endoscopic ultrasound‐guided abscess drainage (EUS‐AD) could effectively treat various abscesses and fluid collections. However, no indications or procedures have been established for EUS‐AD treatments, and studies on its usefulness and safety are insufficient. The present study aimed to evaluate the efficacy and safety of EUS‐AD for treating non‐pancreatic abscesses.

**Methods:**

This retrospective study included 20 patients, aged ≥20 years, who underwent EUS‐AD for an abscess or fluid accumulation in the abdomen or mediastinum, but not the pancreas. Patients were treated at the Kawasaki University General Medical Center between March 2013 and June 2021. All EUS‐AD procedures were performed prior to a percutaneous drainage or surgical drainage.

**Results:**

Among the 20 patients who underwent an EUS‐AD for abscess, 8 (40%) had liver abscesses, 6 (30%) had intraperitoneal abscesses, 3 had (15%) splenic abscesses, 1 (5%) had a mediastinal abscess, 1 (5%) had an iliopsoas abscess (*n* = 1, 5%), and 1 (5%) had an abdominal wall abscess. The technical success rate was 95% (*n* = 19/20). We inserted nasobiliary catheters in 4/20 patients (20%). The clinical success rate was 90% (*n* = 18/20). Two clinical failures required reintervention, and both were treated with percutaneous drainage. Adverse events were observed in 2/20 patients (10%). One patient experienced fever after the procedure, and the other experienced localized peritonitis.

**Conclusion:**

EUS‐AD was effective and safe for abscess removal, particularly when approached from the upper gastrointestinal tract.

## Graphical abstract

EUS‐AD was effective and safe for treating abscesses, particularly when approached from the upper gastrointestinal tract. It should be applied for fluid collections that are larger than 4 cm in diameter and located less than 2cm from the EUS transducer.

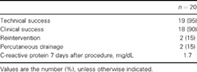

## Introduction

Abscesses and fluid collections occur under various conditions. A liver abscess can be caused by biliary infection; bile leaks can occur during liver surgery or therapeutic endoscopy; a splenic abscess can form because of transcatheter arterial embolization; and a mediastinal abscess can be caused by the accidental ingestion of fish bones. According to previous studies, abdominal or pelvic abscesses occur in 10–30% of individuals with Crohn's disease.^1^, ^2^ These abscesses are generally treated with conservative therapy and antibiotics, percutaneous drainage, or surgery, depending on the patient's general condition and the size and location of the abscess.

Percutaneous drainage is often an effective treatment. Its clinical success was reported to be 78–92.7%.^3^, ^4^, ^5^, ^6^ However, percutaneous drainage is not always feasible because of the location of the abscess. It requires external catheterization, which restricts patients' motion due to pain and is prone to dislocation or self‐extraction. Alternatively, surgical therapy is effective, particularly for large abscesses and abscesses that are difficult to treat with percutaneous drainage because of their proximity to the digestive tract or artery. However, surgical treatment is invasive. Some abscesses are intractable and require a long hospital stay. Therefore, we need a novel, effective method for abscess control.

Recently, studies have shown that endoscopic ultrasound‐guided abscess drainage (EUS‐AD) is effective for treating various abscesses and fluid collections. EUS‐guided drainage was previously used successfully for treating walled‐off necrosis (WON) or pancreatic pseudocysts (PCs) after acute pancreatitis. In 1992, Grimm et al. described the first EUS‐guided drainage for PCs.^7^ In the case of WON, EUS‐guided drainage could achieve an internal fistula in only one step, and the clinical success rate for drainage was 91–97%.^8^, ^9^ Therefore, EUS‐guided drainage is often the first choice for WON. Except for abscesses in the pancreas, encapsulated abscesses such as WON or PCs can be drained through the intestinal tract with EUS. Some studies have shown that EUS‐AD is effective and safe for treating abscesses in various locations, except the pancreas. On the basis of these findings, it is reasonable to assume that abscesses near the digestive tract can be treated through the digestive tract with EUS. However, there are no established indications or procedures for performing EUS‐AD, and its usefulness and safety have not been sufficiently studied. The present retrospective study aimed to evaluate the efficacy and safety of EUS‐AD for treating non‐pancreatic abscesses.

## Materials and methods

This study was a retrospective observational study. Its protocol conformed to the 1975 Helsinki Declaration and was approved by the Institutional Research Ethics Committee (Admission No: 5557‐00).

### Patients

The study was conducted between March 2013 and June 2021 in the Kawasaki University General Medical Center. We included 20 patients, aged ≥20 years, who had undergone an EUS‐AD for an abscess or fluid collection in the abdomen or mediastinum, but not the pancreas. All EUS‐ADs were the first drainage procedure, prior to percutaneous drainage or surgical drainage.

Abscesses or fluid collections were detected with computed tomography (CT), magnetic resonance imaging, abdominal ultrasound, and/or EUS. Endoscopists judged the suitability of the approach based on the abscess size (>4 cm) and the distance from the gastrointestinal tract (<2 cm).

### Procedure

EUS‐AD was performed with a curved linear‐array echo‐endoscope (GF‐UCT260; Olympus Co. Ltd., Tokyo, Japan). The abscess was delineated on EUS, and the aspiration position was located as close to the abscess as possible. Care was taken to avoid puncturing the thorax by checking the X‐ray image, particularly near the esophagogastric junction. Briefly, the abscess was punctured with 19‐gauge needle, and the position of needle tip was checked in the X‐ray and ultrasound images. After confirming that the content of abscess was pus, a 0.025‐in. guidewire was inserted. The needle tract was dilated with a 6‐mm‐diameter dilation balloon. When the wall of abscess was too thick to advance a catheter, electrocautery was used to perform the dilation. After dilation of the fistula, another 0.035‐in. guidewire was inserted into the abscess cavity using an uneven double‐lumen cannula (Piolax Medical Devices, Kanagawa, Japan) to achieve double guidewire placement. Finally, two or more 7 Fr. double pig‐tail stents were placed. When the endoscopist judged it necessary to insert a nasobiliary catheter to monitor the quantity or properties of the pus, only one nasobiliary catheter or one plastic stent and one nasobiliary catheter were inserted. In addition, the insertion of a nasobiliary catheter was also considered in cases where the distance could increase considerably when the abscess shrank. After completing the EUS‐AD, the abscess was checked with CT imaging. With effective drainage, the abscesses became smaller or included air. One plastic stent was removed by endoscopy at 3–6 months after the procedure, and other stents were removed after another 3–6 months. Since a space often remained in the area where the double pig‐tail stents were placed, in order to prevent recurrence of the abscess, we removed the stents one at a time thereby reducing the space gradually.

**Figure 1:**
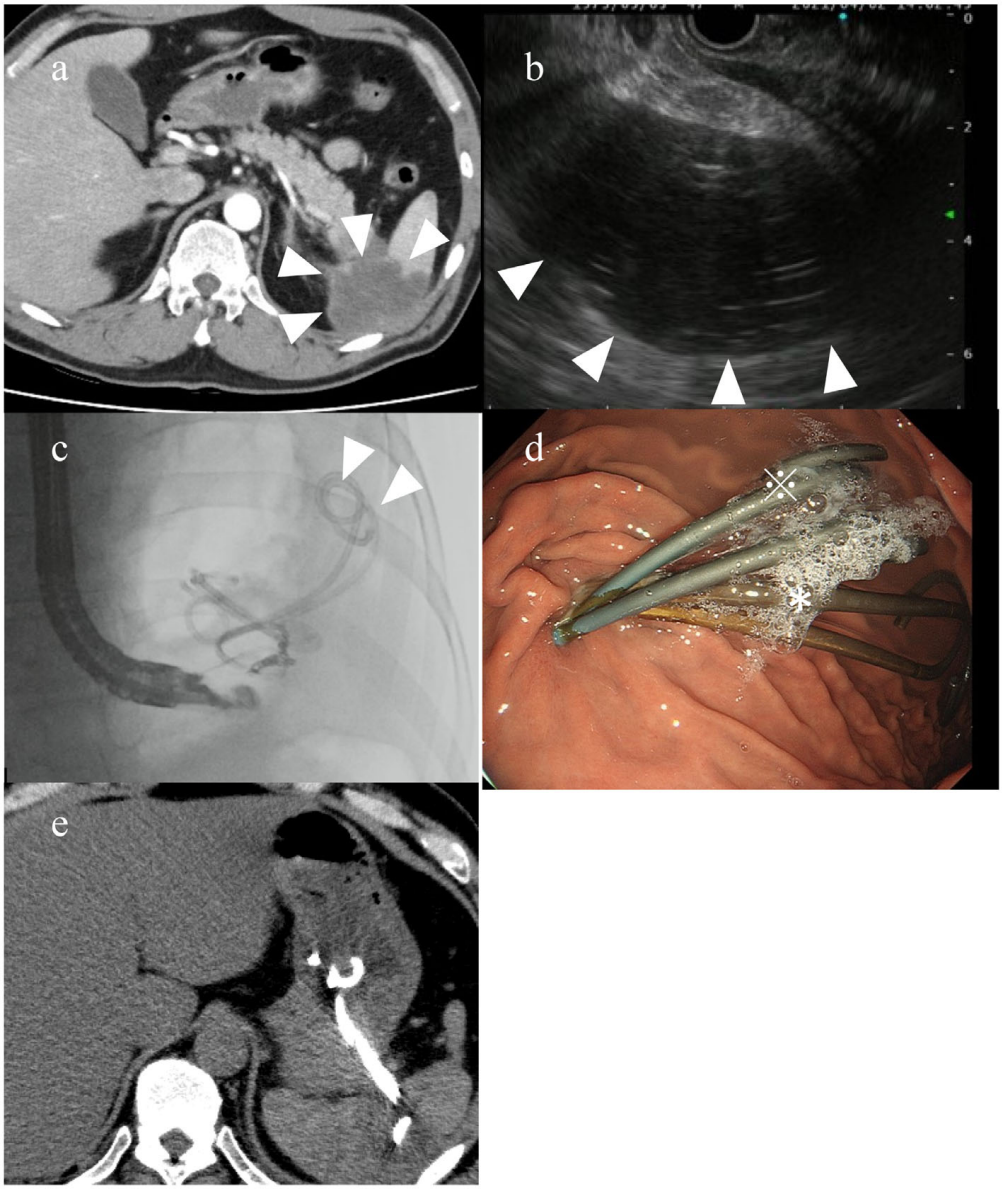
Treatment of a splenic abscess that formed after transcatheter arterial embolization for treating a pseudoaneurysm rupture of the splenic artery. This patient had previously received two plastic stents for draining a pancreatic cyst that formed after acute pancreatitis. (a) Computed tomography (CT) shows a low‐density area in the spleen region, which was considered a splenic abscess (arrowheads). (b) Endoscopic ultrasound image shows a hypoechoic area next to the stomach (arrowheads). (c) We punctured the hypoechoic area from inside the stomach, dilated the fistula with a balloon, and inserted two plastic stents (arrowheads). (d) Endoscopic image inside the stomach shows the two stents inserted for the splenic abscess (*) and two stents that had been inserted previously for a pancreatic cyst (※). (e) CT after EUS‐AD showed two plastic stents from stomach into splenic abscess.

Figure 1 shows the treatment course of a splenic abscess. It occurred after a transcatheter arterial embolization for treating a pseudoaneurysm rupture of the splenic artery. Previously, a pancreatic cyst had developed with acute pancreatitis, and the cyst had been drained with two plastic stents. CT showed a low‐density area in the spleen, which was considered a splenic abscess. We punctured it with a 19‐gauge needle from the stomach, dilated the fistula with a 6‐mm‐diameter dilation balloon, and inserted two plastic stents. One stent in the splenic abscess was removed 3 months after the procedure, and the other stent was removed 6 months after the procedure. After stent removal, the splenic abscess did not recur.

### Statistical analysis

The primary outcomes of this study were the technical and clinical success rates of the EUS‐AD. Technical success was defined as the successful placement of plastic stents or nasobiliary catheters into the abscess, after puncturing the abscess. Clinical success was defined as disappearance of symptoms or inflammation on blood examination without reintervention. The secondary outcome was the incidence of adverse events.

All statistical analyses were performed with IBM SPSS Statistics version 26 (IBM, Armonk, NY).

## Results

### Patient characteristics

Among the 20 patients enrolled (Tables 1, 2), the most common abscess location was the liver (*n* = 8, 40%). The next most common locations were the peritoneum (*n* = 6, 30%), spleen (*n* = 3, 15%), mediastinum (*n* = 1, 5%), iliopsoas muscle (*n* = 1, 5%), and the abdominal wall (*n* = 1, 5%). Two abscesses (10%) were approached with EUS from the esophagus, 15 (75%) from the stomach, and 3 (15%) from duodenum. The median abscess size was 54 mm.

**Table 1:** Baseline characteristics of patients who underwent EUS‐AD for abscesses

Characteristic	Patients, *n* = 20
Age	70.5 (IQR: 66–85)
Sex, male	12 (60)
*Location of abscess*	
Liver abscess	8 (40)
Intraperitoneal abscess	6 (30)
Splenic abscess	3 (15)
Mediastinal abscess	1 (5)
Iliopsoas abscess	1 (5)
Abdominal wall abscess	1 (5)
Abscess size, mm	54 (IQR: 45–90)
C‐reactive protein level before procedure, mg/dL	8.6 (IQR: 3.1–15.0)
*Location of puncture*	
Esophagus	2 (10)
Stomach	15 (75)
Duodenum	3 (15)
Procedure time, min	27.5 (IQR: 24–34)
Length of hospital stay, days	15 (IQR: 11–22)

Values are the number (%) unless otherwise indicated.

IQR, interquartile range.

**Table 2:** Summary of all cases

No.	Age	Sex	Location	Abscess size(mm)	Puncture site	Number of stents	Technical success	Clinical success	Duration of drainage period (days)	Drainage tubes	EUS distance (mm)	CT distance(before procedure) (mm)	CT distance (after procedure) (mm)
1	87	F	Liver	158	Stomach	2	+	+	413	7Fr.10 cm PS	10	0	8
										7Fr.15 cm PS			
2	88	F	Liver	115	Stomach	3	+	+	197	7Fr.7cmPS	12	0	0
										7Fr.7 cm PS			
										7Fr.4 cm PS			
3	88	F	Liver	175	Stomach	2	+	−	32	7Fr.10 cm PS	22	38	51
										7Fr.10 cm PS			
4	72	F	Liver	57	Duodenum	2	+	+	466	7Fr.4 cm PS	0	0	8
										7Fr.4 cm PS			
5	47	M	Spleen	52	Stomach	2	+	+	361	7Fr.7 cm PS	8	7	5
										7Fr.7 cm PS			
6	50	M	Mediastinum	30	Esophagus	2	+	+	14	5Fr.4 cm PS	0	0	0
										5Fr. ENBD			
7	85	M	Liver	45	Stomach	0	−	+	−	−	25	25	−
8	44	M	peritoneum	44	Stomach	1	+	+	377	7Fr.4 cm PS	3	7	12
9	69	M	Liver	50	Stomach	2	+	+	217	7Fr.7 cm PS	5	25	25
										7Fr.7 cm PS			
10	69	M	Liver	61	Duodenum	1	+	+	367	7Fr.7 cm PS	12	26	34
11	69	M	Spleen	46	Stomach	2	+	+	82	7Fr.4 cm PS	8	7	17
										7Fr.4 cm PS			
12	67	F	Peritoneum	40	Stomach	1	+	+	12	7Fr. ENBD	10	28	24
13	81	F	Spleen	83	Stomach	3	+	+	61	7Fr.4 cm PS	0	0	0
										7Fr.4 cm PS			
										7Fr. ENBD			
14	66	M	Peritoneum	42	Esophagus	1	+	+	138	7Fr.4 cm PS	0	0	0
15	64	M	Peritoneum	109	Stomach	1	+	+	5	6Fr. ENBD	5	0	0
16	78	M	Liver	120	Stomach	2	+	+	224	7Fr.4 cm PS	7	15	28
										7Fr.4 cm PS			
17	85	M	Abdominal wall	37	Stomach	1	+	−	65	7Fr.4 cm PS	18	6	6
18	19	F	Peritoneum	56	Stomach	3	+	+	424	7Fr.7 cm PS	0	0	0
										7Fr.4 cm PS			
										7Fr.4 cm PS			
19	79	M	Peritoneum	51	Stomach	2	+	+	110	7Fr.7 cm PS	10	8	19
										7Fr.7 cm PS			
20	94	F	Iliopsoas muscle	63	Duodenum	1	+	+	20	7Fr.7 cm PS	8	38	45

CT, Computed tomography; ENBD, endoscopic nasobiliary drainage tube; EUS, endoscopic ultrasound; PS, plastic stent.

### Outcomes

Table 3 shows the outcomes of this study. The technical success rate was 95% (*n* = 19/20). We inserted nasobiliary catheters in 4/20 patients (20%). The one technical failure was in treating a liver abscess, where the thorax was punctured from the esophagus. Therefore, the liver abscess was aspirated, and no stent or drainage tube was inserted. As a result, the liver abscess improved, with no complications or recurrences. The clinical success rate was 90% (*n* = 18/20 patients) for the first EUS‐AD. Two clinical failures required reinterventions, and both were treated with percutaneous drainage. After reintervention, both abscesses improved. The median hospital stay was 15 days.

**Table 3:** Outcomes of patients that underwent EUS‐AD for abscesses

	*n* = 20
Technical success	19 (95)
Clinical success	18 (90)
Reintervention	2 (15)
Percutaneous drainage	2 (15)
C‐reactive protein 7 days after procedure, mg/dL	1.7

Values are the number (%), unless otherwise indicated.

Adverse events were observed in 2/20 patients (10%). One patient developed fever after the procedure, and the other developed localized peritonitis. Both cases were mild, and they improved without intervention or therapy. An abscess recurred in one patient (5%), and it improved with a second EUS‐AD.

## Discussion

This study demonstrates that EUS‐AD is effective and safe for treating abscesses. Previous studies have reported success rates of 77–100% for EUS‐AD.^3^, ^10^, ^11^ In the present study, the EUS‐AD success rate was 90%, which is comparable to those of previous studies. In all our patients, the abscess puncture was performed from the upper gastrointestinal tract because no pelvic abscesses were observed over the study period. Many previous reports have reported that a transrectal EUS‐AD was effective for treating pelvic abscesses, and some studies have shown that a transesophageal EUS‐AD was effective for treating mediastinal abscesses.

We considered that EUS‐AD was applicable not only to abscesses near the upper gastrointestinal tract but also to any abscesses within puncturable distance. In determining the suitability of EUS‐AD, we considered that the most important factor was distance, rather than location. Abscesses that are far from the digestive tract present difficulties for stent insertion, and the possibility of stent migration increases during the procedure. In addition, after stent placement, as the abscess shrinks, the distance between the puncture site and the abscess increases. Therefore, EUS‐AD should be considered suitable only in cases where the abscess is close to the digestive tract. Moreover, abscesses <4 cm in diameter can be improved with antibiotics alone.^12^ Consequently, drainage therapy should be considered only for abscesses >4 cm in diameter. Similarly, previous studies have shown that EUS‐AD for the pelvic abscess was effective and safe for fluid collections greater than 4 cm and less than 2 cm of the EUS transducer.^13^, ^14^ Ulla‐Rocha et al. reported that the distance between the abdominal abscess and the EUS transducer was less than 1 cm.^15^ In the present study, while many cases had a distance of less than 1 cm, there were two cases of hepatic abscesses where the distance between the abscess and the EUS transducer was more than 2 cm. However, the distance between the liver and the EUS transducer was less than 2 cm. We considered that it was safe when the distance between the EUS transducer and the abscess or the organ was less than 2 cm. These criteria may also be applicable to abscesses.

In the present study, plastic stents or nasobiliary catheters were used, similar to previous studies. Nasobiliary catheters have the advantage of allowing one to monitor the drained pus and possibly rinsing out the abscess cavity. However, an external catheter can cause distress to patients, which induces dislocation or self‐extraction. Therefore, we preferred plastic stents to external catheterization. Alternatively, some reports have shown that metal stents were effective.^3^, ^16^, ^17^, ^18^ For example, Ogura et al. showed that EUS‐AD with a metal stent for a liver abscess had a clinical success rate comparable to that achieved with percutaneous drainage (100% *vs* 89%, *P* = 0.34).^3^ Metal stents have a larger diameter than plastic stents. Therefore, decompression can be done rapidly and effectively. Recently, studies have shown that the lumen‐apposing metal stent (LAMS) was effective for treating various abscesses.^16^, ^17^, ^18^ LAMS has bilateral anchor flanges, which can stabilize the stent position and prevent stent dislocation. Itoi et al. showed that LAMS was effective for treating pancreatic PCs and draining the gallbladder.^19^ However, both the conventional metal stent and LAMS are expensive. Most abscesses are predominantly filled with liquid, which can be adequately drained with plastic stents. In our group, a metal stent or LAMS is considered necessary only for abscesses that are refractory to plastic stenting.

In the present study, we observed adverse events in only 10% of patients, and none of these was severe. In previous studies, the adverse event rates were 0–22.2%.^14^, ^20^, ^21^ The incidence of adverse events with EUS‐AD was comparable to that observed with percutaneous drainage.^3^, ^20^ Previous studies have reported that the adverse events associated with EUS‐AD included bleeding, stent dislocation, or perforation, which was sometimes severe or fatal. Since intraperitoneal stent migration can require surgery, we have to pay special attention to avoid it. For the procedures described in the present study, we avoided EUS‐AD for abscesses that were located at a large distance from digestive tract. In addition, nasobiliary catheters were effective for cases in which the insertion of plastic stents was considered difficult.

This study had several limitations. The study design was retrospective, and the design was a single‐arm study. In addition, the indications for treatment were determined with reference to imaging studies in our hospital. We had no definitive criteria for inclusion, which might be considered a selection bias. Finally, we only included EUS‐ADs performed from the upper gastrointestinal tract. Therefore, we could not evaluate the efficacy of EUS‐AD performed from the rectum.

In conclusion, our findings demonstrate that EUS‐AD is effective and safe for treating abscesses, particularly when approached from the upper gastrointestinal tract. However, this procedure can cause severe adverse events, particularly stent migration. To avoid adverse events, indications must be carefully considered, and criteria should be based on previous reports. Based on those criteria, EUS‐AD should be applied for fluid collections that are larger than 4 cm in diameter and located less than 2 cm from the EUS transducer.
